# An Efficient Mosaic Algorithm Considering Seasonal Variation: Application to KOMPSAT-2 Satellite Images

**DOI:** 10.3390/s150305649

**Published:** 2015-03-09

**Authors:** Jaewon Choi, Hyung-Sup Jung, Sang-Ho Yun

**Affiliations:** 1 The Department of Geoinformatics, The University of Seoul, Seoul 130-743, Korea; E-Mail: jwchoi74@uos.ac.kr; 2 The Jet Propulsion Laboratory, California Institute of Technology, Pasadena, CA 91109, USA; E-Mail: Sang-Ho.Yun@jpl.nasa.gov

**Keywords:** high-resolution optical satellite imagery, Kompsat-2, seasonal characteristics, seamlines, feathering algorithm

## Abstract

As the aerospace industry grows, images obtained from Earth observation satellites have been successfully used in various fields. Specifically, the demand for a high-resolution (HR) optical images is gradually increasing, and hence the generation of a high-quality mosaic image is being magnified as an interesting issue. In this paper, we have proposed an efficient mosaic algorithm for HR optical images that are significantly different due to seasonal change. The algorithm includes main steps such as: (1) seamline extraction from gradient magnitude and seam images; (2) histogram matching; and (3) image feathering. Eleven Kompsat-2 images characterized by seasonal variations are used for the performance validation of the proposed method. The results of the performance test show that the proposed method effectively mosaics Kompsat-2 adjacent images including severe seasonal changes. Moreover, the results reveal that the proposed method is applicable to HR optic images such as GeoEye, IKONOS, QuickBird, RapidEye, SPOT, WorldView, *etc.*

## 1. Introduction

Korea is enforcing the 1st National Aerospace Development Mid & Long Term Plan from 1995 to 2015. Following this plan, Korea is developing a systematic strategy and a multipurpose satellite series (e.g., Kompsat 1, 2, 3 and 5), which have high-spatial-resolution optical sensors and X-band Synthetic Aperture Radar (SAR) sensors, for comprehensive risk assessments and to form a better-informed land use plan. The Ministry of Education (MOE) is contributing significantly to Korea’s development of the national aerospace policy and the utilization of high quality information from satellite remote sensing. The Korea Aerospace Research Institute (KARI, Daejeon, Korea), an affiliated institute of the Korea Research Council of Fundamental Science & Technology, is actively providing high-performance, cutting-edge remote sensing technology to domestic and foreign research scientists. Recently, the successful launch of the Kompsat series (e.g., Kompsat 3 and 5), which have sub-meter spatial resolutions and X-band SAR sensors, increased the supply of data to international and federal, regional, state and local users. Moreover, high-end mosaicked Kompsat series images, including full coverage of the Republic of Korea, are required for the development of better mosaic algorithms for easy use and better adaptation to seasonal changes (e.g., the four seasons—spring, summer, fall and winter). The Ministry Of Security and Public Administration (MOSPA, Seoul, Korea) establishes the integrated disaster response system and safety network, formulates policies for the protection of nation’s critical infrastructure, oversees plans for the creation of a safe pedestrian environment and safety zones for children, plans and coordinates policies for emergency preparedness and oversees affairs related to national resource mobilization. These applications widely utilize mosaicked Kompsat series images for safety management.

A mosaic of optical satellite remote sensing images (e.g., from high spatial resolution to low spatial resolution) can be explained simply as stitching two or more orthorectified satellite images with an overlapping area if the images from the satellite do not include atmospheric effects. However, optical satellite images often include haze (e.g., water vapor and aerosol particles). Creating mosaics generally requires several conditions that should be common across input images; map projection (e.g., geographic lat/lon and UTM), pixel size and rotation. To create a mosaic of two or more optical satellite remote sensing images, we geometrically correct the raw optical remote sensing dataset to a known map coordinates system (e.g., geographic coordinates system or projected coordinates system) and also have to preprocess the atmospheric corrections (e.g., image-based model, empirical line model and atmospheric condition model).

The image mosaic algorithms were studied by interested researchers or groups for developing advanced algorithms. The numerous mosaic algorithm research studies over the past 10 years can be summarized as: (1) digital image mosaic algorithms [1,2,3] which use digital images and video data; (2) urban and rural area mosaic algorithms, which are based on aerial photography [4,5,6,7,8,9,10]; and (3) optical and radar satellite images, which cover areas from local to global using mosaic algorithms [11,12,13,14]. The image processing field is mostly studying optimized scale-and-stretch for image resizing [1,2,3] and are deciding seamline using digital images acquired from digital cameras and camcorders. The high-resolution image processing field, which is based on aerial photographs, is focusing on Bidirectional Reflectance Distribution Function (BRDF) correction [7,8], empirical models for radiometric calibration of digital aerial frame mosaic [8], and tracking of vector roads for the determination of seams in aerial image mosaics [9]. The mosaic algorithms from satellite image can be divided into two groups: optical image and Synthetic Aperture Radar (SAR) images. The optical images (e.g., high-resolution images, multi-spectral images or hyper-spectral images) based on spectral analysis focus on radiometric normalization, compositing and quality control [11] for an operational mosaicing of high resolution images over large areas. For SAR images (e.g., X-, C- and L-band SAR images), Advanced Land Observing Satellite (ALOS), Phased Arrayed L-band Synthetic Aperture Radar (PALSAR) and TerraSAR-X image were studied. In most previous studies, mosaic tests have been performed by using images whose surface reflectivity patterns are very similar. However, it is very important to consider apparent seasonal changes in order to mosaic images obtained from different seasons because we have difficulty in acquiring high-resolution optical images in the same season due to weather conditions. Moreover, most previous studies for mosaic algorithms have focused on the extraction of an optimal seamline in an overlapped area of adjacent images.

The objective of this research is to propose an optimal mosaic procedure for the mosaic of images obtained from different seasons through finding the combination of suitable functions. Using eleven high-resolution Kompsat-2 satellite images, we demonstrate the performance of our algorithm in the presence of seasonal changes in mountainous terrains (mountains cover 70% of Korea). In this research, we suggest an optimal combination of specific algorithms such as masking images, extracting image coordinates, extracting image overlay areas, extracting image seamlines, matching image histograms, and feathering images.

## 2. Data

Kompsat-2 was developed by KARI to provide multi-purpose support (e.g., surveillance of large scale disasters, acquisition of independent, high-resolution images for GIS, survey of natural resources and continuation of satellite Earth observation). There are two products levels for KOMPSAT-2 image data: Level 1R product and Level 1G product. All products are provided as a bundle (pan + 4 multi-spectral) or as a pan-sharpened. Level 1R are the products corrected for radiometric and sensor distortions. Level 1G are the products corrected for geometric distortions and projected to UTM. Table 1 shows the specifications and primary uses of Kompsat-2.

**Table 1:** Kompsat-2 specifications and primary uses.

Specifications	Kompsat-2
Date of Launch	28 July 2006
Orbit type	Sun-synchronous
Repeat Cycle	14 day
Resolution	1 m (panchromatic), 4 m (multispectral)
Swath Width	15 km
Pan	500–900 nm (locate, identify, measure surface features, objects primarily)
MS1 (blue)	450–520 nm (mapping shallow water, differentiating soil from vegetation)
MS2 (green)	520–600 nm (differentiating vegetation by health)
MS3 (red)	630–690 nm (differentiating vegetation by species)
MS4 (near-infrared)	760–900 nm (mapping vegetation vigor/health, differentiating vegetation by species)

**Figure 1:**
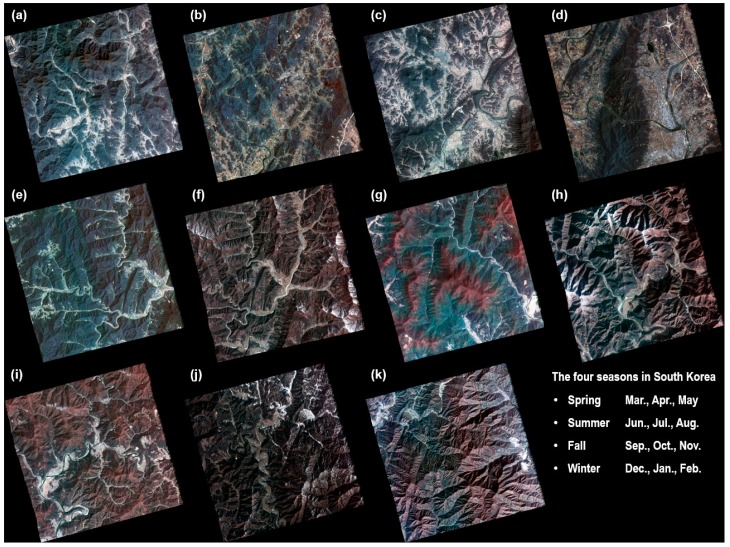
The tested Kompsat-2 images, including the seasonal and topographical characteristics. (**a**) 23 October 2009; (**b**) 23 October 2009; (**c**) 18 May 2009; (**d**) 18 May 2009; (**e**) 15 August 2009; (**f**) 16 March 2008; (**g**) 3 May 2008; (**h**) 28 December 2009; (**i**) 15 April 2008; (**j**) 5 January 2009; (**k**) 7 November 2009.

In this study, we used cloud-free Kompsat-2 image data with pan-sharpened and Level 1G to apply the proposed mosaic algorithm. These images cover 35°–37° latitude and 127°–128° longitude and mostly lie in the southern part of Korea Peninsula. The data are composed of 11 images (Figure 1), including four seasonal and topographical characteristics, difficult conditions for mosaicing. The Kompsat-2 images clearly show snow cover in certain mountain areas. Based on the seasonal changes, the vegetation in the Kompsat-2 images were distinguished as healthy, stressed, or mixed. The solar azimuth angles (SAA) for the acquired Kompsat-2 images are mostly in the range of 82°–87°. Additionally, some Kompsat-2 images show 211, 257 or 33 degrees (Table 2).

**Table 2:** Characteristics of the tested Kompsat-2 images.

Scenes	Date	SAA (deg.)	Lat. (deg.)	Long. (deg.)
Scene (a)	23 October 2009	87.11	35.34	127.15
Scene (b)	23 October 2009	87.11	35.47	127.11
Scene (c)	18 May 2009	211.43	35.34	127.27
Scene (d)	18 May 2009	211.43	35.47	127.23
Scene (e)	15 August 2009	92.23	37.47	128.36
Scene (f)	16 March 2008	257.93	37.47	128.42
Scene (g)	3 May 2008	89.00	37.47	128.57
Scene (h)	28 December 2009	33.54	37.47	128.66
Scene (i)	15 April 2008	82.16	37.47	128.71
Scene (j)	5 January 2009	83.75	37.47	128.86
Scene (k)	7 November 2009	84.46	37.47	128.96

## 3. Methodology

**Figure 2:**
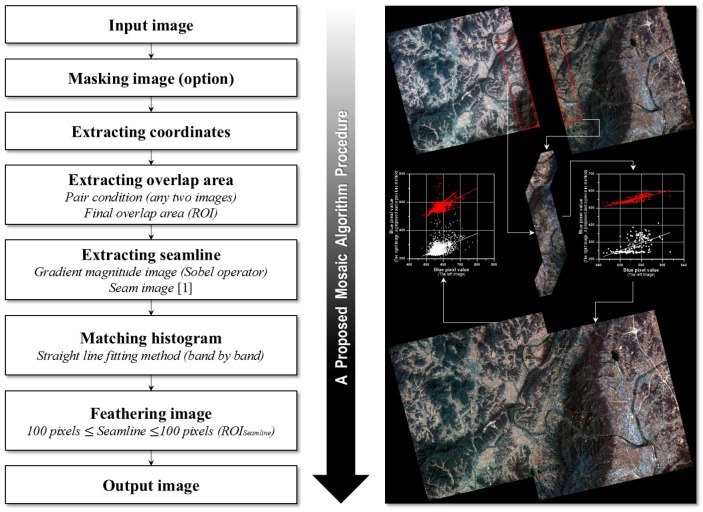
Flowchart of the proposed mosaic algorithm procedure.

A mosaic algorithm was specially designed for high-resolution, multispectral Kompsat-2 images. This algorithm is based on several assumptions described in Section 3.1. It describes an optimal mosaic procedure through finding the combination of suitable functions. Figure 2 shows a flowchart of the proposed mosaic algorithm. In the following section, the detailed procedures are explained.

### 3.1. Assumptions

In this study, we defined three assumptions for the creation of an optimum mosaic product from Kompsat-2 images, and these assumptions are expressed by the following statements:

(1) We only consider orthorectified and pan-sharpened true color Kompsat-2 images that are cloud free.
(2) We do not consider radiometric and atmospheric corrections for the Kompsat-2 images.
(3) Almost all of the image textures from an overlapped area of adjacent Kompsat-2 images have minimal change.

### 3.2. Masking Images

An orthorectified, pan-sharpened high-resolution optical satellite image might have false-color pixel errors along the image boundary. These unintentional false-color pixel errors might occur in an image overlap area if we use these images in a mosaic. If we do not remove these errors, we might obtain low quality mosaic images when we apply the mosaic procedures, such as extracting seamlines, matching histograms, and feathering images. The unintentional false-color pixel errors along an image boundary in a target image can easily be removed using a mask image. The image masking algorithm can generally be applied to a target image by creating a mask image with values of 0 or 1, where 0 and 1 are exclusion and inclusion values, respectively. A mask image has to exactly match the target image information (e.g., map projection, datum, pixel size and tie point) and can remove a specific region in a target image using multiplication.

### 3.3. Extracting Coordinates

Various satellite images come in different data formats, depending on the distribution agency. One of the typical image data formats is the GeoTIFF format, which consists of tiff and tfw file formats. A tfw file format has useful information, including an upper left coordinates and pixel size, and a tiff file format provides digital information about the RGB color values. The Kompsat-2 image data format is the GeoTIFF format, and the extracted UTM coordinates information was used to compute an adjacency overlap area for the Kompsat-2 images. If we know the UTM coordinates of the upper left corner from the tfw file format, the UTM coordinates of the whole image can be calculated by ∆*x* and ∆*y* because the pixel size of orthorectified and pan-sharpened Kompsat-2 image with the UTM coordinates has equal intervals along the x- and y-direction. Let *X_UTM_* and *Y_UTM_* represent the UTM coordinates values for x- and y-direction at any image coordinates (*x*, *y*). Then the UTM coordinates information for each pixel in a Kompsat-2 image (e.g., *m* × *n*) can be expressed by the following formula:

(1) $$\begin{array}{l} X_{UTM}=\{x|x_{i}=\;x_{UL}+\;i\;\times \Delta x,\;i=1,\;\cdots ,\;m\}\\ Y_{UTM}=\{y|y_{i}=\;y_{UL}+\;i\;\times \Delta y,\;j=1,\;\cdots ,\;n\} \end{array}$$

where *X_UL_* and *Y_UL_* are the upper left coordinates and ∆*x* and ∆*y* are the pixel size of the row and column, respectively.

### 3.4. Extracting the Overlap Area

Computing the overlap area of the input image data using extracted coordinates is important because a false extracted overlap area will directly or indirectly influence the next procedure (e.g., extracting the seamlines, matching the histograms and feathering the images). First, the adjacent images have to be paired before computing an overlap area from the input image data. If we have a number of input images, then the image pair can easily be decided using information from the tfw file and the swath width of Kompsat-2. Let a pair of any input image data represent *Pair_image_* which is the set of true and false, and let *f*1*_RGB_*(*x*, *y*) and *f*2*_RGB_*(*x*, *y*) represent the value of an image pixel of 1st and 2nd input image data at any image coordinates (*x*, *y*), and let the value of an image pixel at any image coordinates (*x*, *y*) includes UTM coordinates and RGB values. Then a pair condition for any two input image data can be expressed using the following formula:

(2) $$Pair_{image}=\{\begin{array}{l} true\;if\;f1_{RGB}(x,y)\cap f2_{RGB}(x,y)\neq \varphi \\ false\;otherwise \end{array}$$

If we have already chosen an image pair, then an overlap area from adjacent images can be computed by comparing the extracted coordinates that were already mentioned in Section 3.3. As a major consideration, an image overlap area should include a calculated Root Mean Square Error (RMSE) value using an orthorectification process of Kompsat-2. Let an extracted overlap area represent *OA_image_*(*x*, *y*) which is the value of an image pixel at any image coordinates (*x*, *y*). Then an image overlap area can be represented as a proper subset that can be expressed using the following:

(3) $$OA_{image}(x,y)=f1_{RGB}(x,y)\cap f2_{RGB}(x,y)\;if\;f1_{RGB}(x,y)\cap f2_{RGB}(x,y)\neq \varphi$$

An extracted overlap area was created as a subset for deriving efficient seamlines. Forty-five coordinates with regular intervals from the initial overlap area were computed to obtain an appropriate subset. Let a subset of *OA_image_*(*x*, *y*) represent *SOA_image_*(*x*, *y*) which is the value of an image pixel at any image coordinates (*x*, *y*). The members of *SOA_image_* can be expressed as follows:

(4) $$SOA_{image}=\{a_{1},\;a_{2},\;a_{3},\;\cdots \;a_{45}\}$$

Let a region of interest (*ROI*) from *SOA_image_* represent *ROI* which pixels are included in the region. Then:

(5) $$\begin{array}{c} ROI\subseteq OA_{image}\\ ROI=\{a_{4},\;a_{5},\;a_{10},\;a_{12},\;a_{14},\;a_{32},\;a_{34},\;a_{36},\;a_{41},\;a_{42}\} \end{array}$$

**Figure 3:**
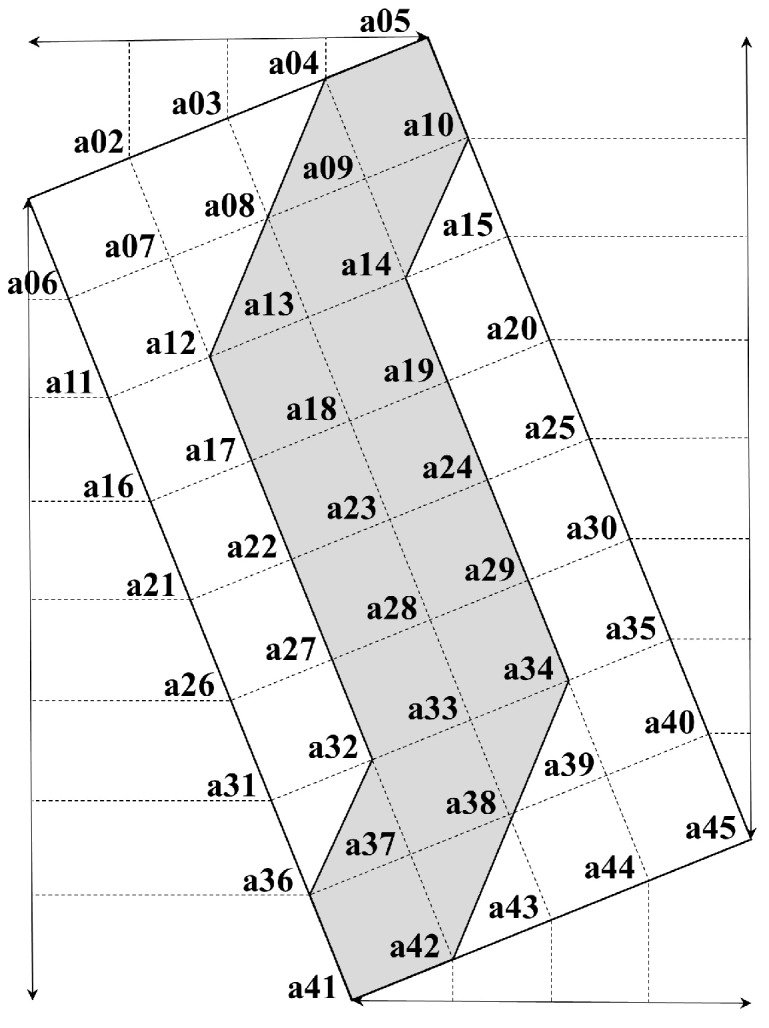
The method for extracting the final overlap area for any two adjacent images.

To calculate the members of *ROI*, Let *a*, *b*, *c*, *d*, *e*, *f*, *g*, *h*, *i* and *j* are variable with floating-point data type. If we know 4 point such as *a*_1_, *a*_5_, *a*_41_ and *a*_45_ (see Figure 3) then:

(6) $$\begin{array}{l} a=(a_{5}(x)-a_{1}(x))/4\;b=(a_{5}(y)-a_{1}(y))/4\\ c=(a_{45}(x)-a_{5}(x))/8\;d=(a_{5}(y)-a_{45}(y))/8\\ e=(a_{15}(x)-a_{11}(x))/4\;f=(a_{15}(y))-a_{11}(y))/4\\ g=(a_{35}(x)-a_{31}(x))/4\;h=(a_{35}(y)-a_{31}(y))/4\\ i=(a_{41}(x)-a_{1}(x))/4\;j=(a_{1}(y)-a_{41}(y))/4\\ a_{4}(x)=a_{1}(x)+3a\;a_{4}(y)=a_{1}(y)+3b\\ a_{10}(x)=a_{5}(x)+c\;a_{10}(y)=a_{5}(y)-d\\ a_{11}(x)=a_{1}(x)+i\;a_{11}(y)=a_{1}(y)-j\\ a_{12}(x)=(a_{15}(x)-a_{11}(x))/4+a_{11}(x)\;a_{12}(y)=(a_{15}(y)-a_{11}(y))/4+a_{11}(y)\\ a_{14}(x)=a_{11}(x)+3e\;a_{14}(y)=a_{15}(x)-f\\ a_{15}(x)=a_{5}(x)+2c\;a_{15}(y)=a_{5}(y)-2d\\ a_{31}(x)=a_{1}(x)+3i\;a_{31}(y)=a_{1}(y)-3j\\ a_{32}(x)=(a_{35}(x)-a_{31}(x))/4+a_{31}(x)\;a_{32}(y)=(a_{35}(y)-a_{31}(y))/4+a_{31}(y)\\ a_{35}(x)=a_{5}(x)+6c\;a_{35}(y)=a_{5}(y)-6d\\ a_{34}(x)=a_{31}(x)+3g\;a_{34}(y)=a_{35}(y)-h\\ a_{36}(x)=a_{41}(x)-(a_{41}(x)-a_{1}(x))/8\;a_{36}(x)=(a_{1}(y)-a_{41}(y))/8+a_{41}(y)\\ a_{42}(x)=(a_{45}(x)-a_{41}(x))/4+a_{41}(x)\;a_{42}(y)=(a_{45}(y)-a_{41}(y))/4+a_{41}(y) \end{array}$$

Let *FOA_image_*(*x*, *y*) represent the value of an image pixel at any image coordinates (*x*, *y*). Then the final overlap area for the next mosaic procedure can be joined into a subset using the following formula:

(7) $$\begin{array}{c} FOA_{image}\subseteq OA_{image}\\ FOA_{image}(x,y)=ROI[OA_{image}] \end{array}$$

Figure 3 shows an example of how to divide regular intervals to get the final overlap area.

### 3.5. Extracting Seamline

A seamline can be calculated using a 2-step procedure (e.g., creating a gradient magnitude image and a seam image). A gradient magnitude image can be computed by convolution between the final overlap area image and kernels, and provides the evidence (e.g., the emphasized edges) to find a seamline. The gradient operator is defined as the following:

(8) $$\nabla =\left(\frac{\partial }{\partial x_{1}},\;\frac{\partial }{\partial x_{2}},\;\cdots \;\frac{\partial }{\partial x_{n}}\right)$$

Typically, a gradient magnitude image can be calculated from the final overlap area image by applying a kernel operator as in the following equation:

(9) $$g=\sqrt{{\left(k_{1}\times f\right)}^{2}+{\left(k_{2}\times f\right)}^{2}}\;or\;g=\left|k_{1}\times f\right|+\left|k_{2}\times f\right|$$

where × represents convolution.

In this study, the Sobel operator was used to compute gradient image from final overlap area images, which can be given by the following formula:

(10) $$\begin{array}{c} k_{x}=\left[\begin{array}{lll} -1 & 0 & 1\\ -2 & 0 & 2\\ -1 & 0 & 1 \end{array}\right],\;k_{y}=\left[\begin{array}{lll} 1 & 2 & 1\\ 0 & 0 & 0\\ -1 & -2 & -1 \end{array}\right]\\ G_{R}=\left|k_{x}\times FOA_{R}\right|+\left|k_{y}\times FOA_{R}\right|\\ G_{G}=\left|k_{x}\times FOA_{G}+\left|k_{y}\times FOA_{G}\right|\right|\\ G_{B}=\left|k_{x}\times FOA_{B}\right|+\left|k_{y}\times FOA_{B}\right|\\ G_{image}=\frac{1}{3}(G_{R}+G_{G}+G_{B}) \end{array}$$

where *G_R_*, *G_G_* and *G_B_* are gradient magnitude images of the red, green and blue bands.

A seam image can be calculated using a computed gradient magnitude image. Generally, a gradient magnitude image emphasizes edges, but the calculation of seamline cannot easily be decided by this information even if we compute a threshold to find ideal edge pixels on a gradient magnitude image. Avidan and Shamir [1] discussed a solution for determining seam images, and the following formula was suggested:

(11) $$Seam_{image}=\sum_{i=1}^{m}\sum_{j=1}^{n}G_{image}(i,j)+\min(G_{image}(i-1,j-1),\;G_{image}(i-1,j),\;G_{image}(i-1,j+1))$$

A new seam line segment is drawn by choosing the minimum value from three adjacent pixels in the *i* direction and connecting it to a previously chosen pixel. Interacting this process defines a seam curve.

### 3.6. Matching Histogram

The histogram matching algorithm [15] is a similar method for adjusting the color of two images with different colors using a reference image histogram. This method is a necessary procedure for making high quality mosaic products, and can be calculated using the equation of the straight line that minimizes the sum of the squares of the residuals. Therefore, the equation of the straight line is composed of an intercept (e.g., a) and a slope (e.g., b). Generally, a true color image has to calculate three equations per pair because it is composed of three layers, *i.e.*, the red, green and blue bands. In this study, we used the straight line fitting method instead of traditional histogram matching algorithm. The equation of the linear fit and the vertical deviations, *R^2^*, of a set of *n* data points can be expressed by the following formula:

(12) $$\begin{array}{c} \left[\begin{array}{l} a\\ b \end{array}\right]=\frac{1}{n\sum_{i=1}^{n}x_{i}^{2}-{\left(\sum_{i=1}^{n}x_{i}\right)}^{2}}\left[\begin{array}{l} \sum_{i=1}^{n}y_{i}\sum_{i=1}^{n}x_{i}^{2}-\sum_{i=1}^{n}x_{i}\sum_{i=1}^{n}x_{i}y_{i}\\ n\sum_{i=1}^{n}x_{i}y_{i}-\sum_{i=1}^{n}x_{i}\sum_{i=1}^{n}y_{i} \end{array}\right]\\ if\;f(a,b)=a+bx\;and\;R^{2}=\sum {\left[y_{i}-f(x_{i},\;x_{i},\;x_{i},\;\cdots ,\;x_{i},\;)\right]}^{2} \end{array}$$

### 3.7. Feathering Image

**Figure 4:**
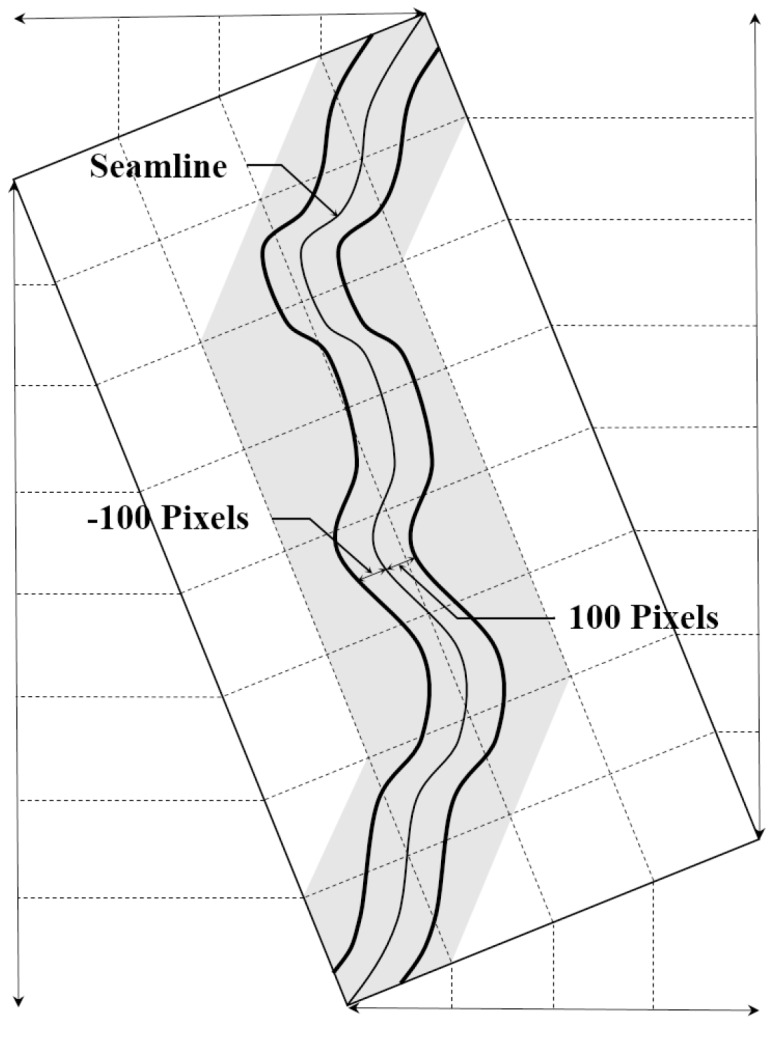
The subset procedure, each 100 pixels to the left and right of the center of each seamline.

Although showing the same area, the satellite images obtained at different times might show different textures. If the acquisition times of any two satellite images are short or continuous, then the change in texture of an image overlap area might show minimal or no change. The image feathering technique could be used to merge a natural image using any two images. One can reduce the level of discontinuity along the image boundary by controlling the transparency in the cross direction. Each image overlap area is 200 pixels wide (Figure 4). Let *Seam_line_*(*x*, *y*) represent the value of an image pixel at any image coordinates (*x*, *y*), and let buffer polygon around input *Seam_line_*(*x*, *y*) to a specified distance with 100 pixels represent *ROI_seamline_*, which pixels are included in the region, and let *OA_image_*(*x*, *y*) of *f*1*_RGB_*(*x*, *y*) represent *OA*1*_image_*(*x*, *y*) which is the value of an image pixel at any image coordinates (*x*, *y*), and let *OA_image_*(*x*, *y*) of *f*2*_RGB_*(*x*, *y*) represent *OA*2*_image_*(*x*, *y*) which is the value of an image pixel at any image coordinates (*x*, *y*), then the feathering images can be expressed using following formulas:

(13) $$\begin{array}{c} LOA_{image}(x,y)=ROI_{seamlline}[OA1_{image}(x,y)]\\ ROA_{image}(x,y)=ROI_{seamlline}[OA2_{image}(x,y)]\\ F_{image}=\alpha LOA_{image}+(1-\alpha )ROA_{image} \end{array}$$

where α is a set of weighting values.

## 4. Results

**Figure 5:**
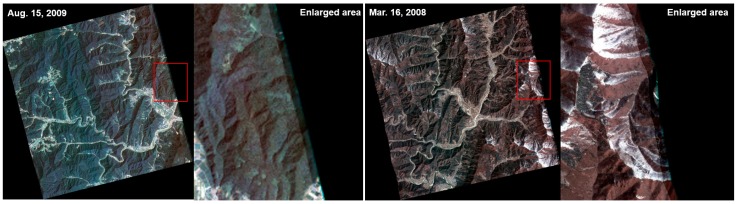
The false color values along the right side of the Kompsat-2 images.

In this study, 11 images from Kompsat-2 with the GeoTIFF file format were used as the basic data to test the proposed, mosaic algorithm that was discussed in Section 3, and these data showed characteristics of the four seasons. All of the images were orthorectified and pan-sharpened Kompsat-2 and included false color values along the right side (see Figure 5). To remove these errors, a total of 11 mask images were created and given values of either 0 or 1. These errors were removed using multiplication between the input data and mask image. To compute the overlap area of image pairs, the coordinates of each Kompsat-2 image were calculated. The useful information, such as the “pixel size” and “map coordinates of the tie point” was extracted to calculate the coordinates from the tfw file.

Using the extracted information and the row and column values, the coordinates of each Kompsat-2 image were computed. Image pairs of adjacent Kompsat-2 images were determined using the calculated coordinates, and a total of eight image pairs were formed from the 11 Kompsat-2 images (Table 3).

**Table 3:** Pairs of the tested Kompsat-2 images.

Pairs	Left Image	Acquired Date	Right Image	Acquired Date
Pair (1)	Scene (a)	23 October 2009	Scene (b)	23 October 2009
Pair (2)	Scene (c)	18 May 2009	Scene (d)	18 May 2009
Pair (3)	Scene (e)	15 August 2009	Scene (f)	16 March 2008
Pair (4)	Scene (f)	16 March 2008	Scene (g)	3 May 2008
Pair (5)	Scene (g)	3 May 2008	Scene (h)	28 December 2009
Pair (6)	Scene (h)	28 December 2009	Scene (i)	15 April 2008
Pair (7)	Scene (i)	15 April 2008	Scene (j)	5 January 2009
Pair (8)	Scene (j)	5 January 2009	Scene (k)	7 November 2009

**Figure 6:**
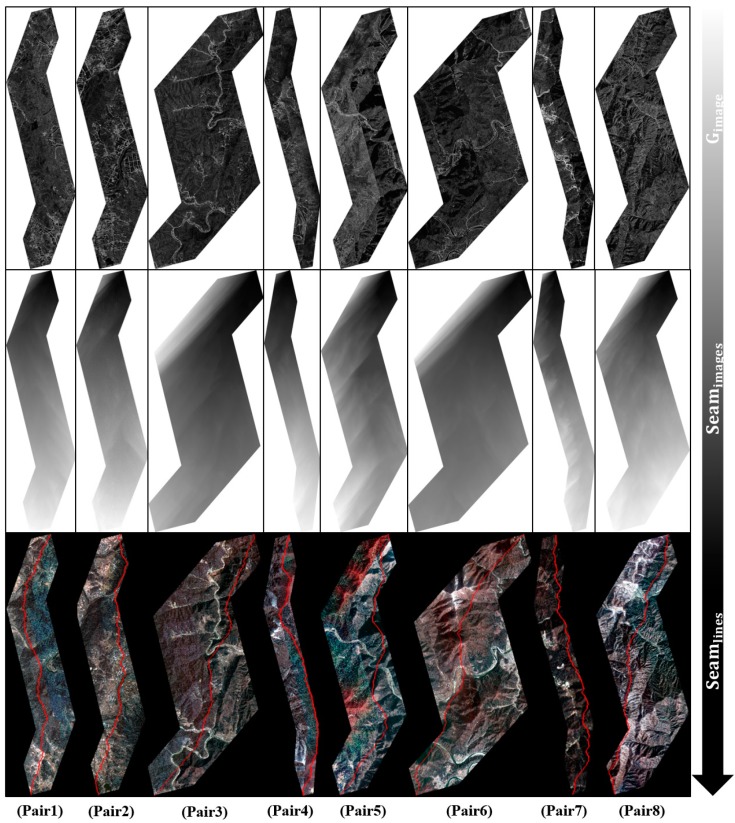
The results of each procedure, *i.e.*, the gradient magnitude image, seam image and seamline.

Additionally, the overlap area of an image pair was determined using the calculated coordinates. If the coordinates of any left and right Kompsat-2 images exactly have an intersection, then it was used as an initial overlap area, and the final overlap area was calculated using the method illustrated in Figure 3. To determine the seamlines, gradient magnitude images and seam images were calculated from the final overlap area (Figure 6).

**Figure 7:**
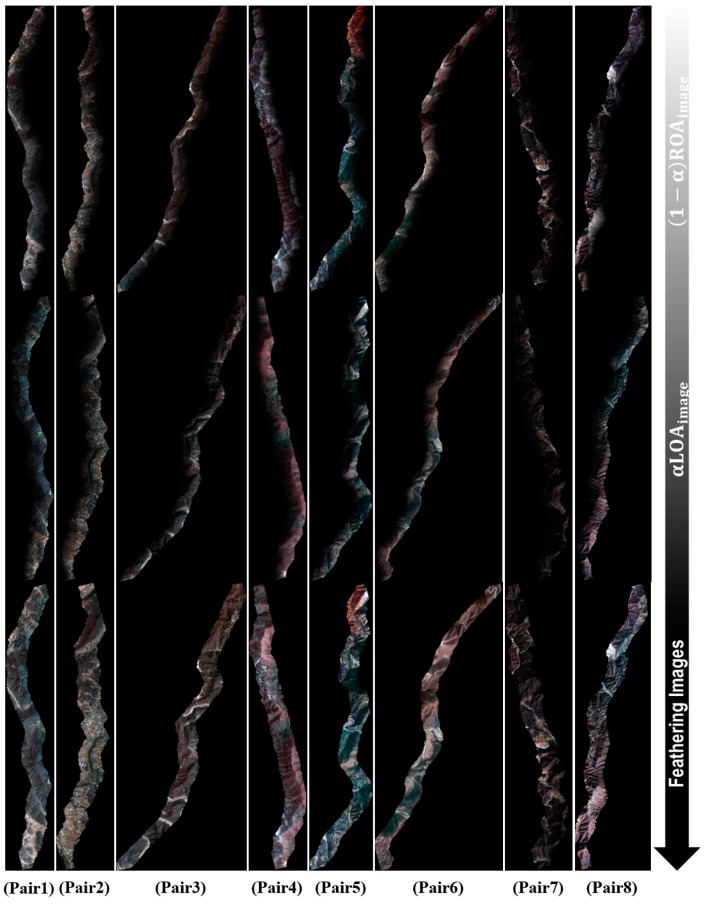
The products of each step and the results of feathering the image.

A gradient magnitude image was computed by convoluting each band (e.g., R, G, and B) and the Sobel operators. A seam image was extracted using a computed gradient magnitude image, and a total of eight seamlines were calculated along the minimum values. For color matching of the mosaic image, a total of 24 straight line equations were calculated from the eight overlap areas (Table 4). These equations were used to adjust the color of the final mosaic images. Using the extracted seamlines, image feathering was applied to the overlap area as the final step of the procedure (Figure 7).

**Table 4:** Calculated Equations of the Straight Lines for the Matching Histogram.

Pairs	Equation of Straight Line		
	Red Band	Green Band	Blue Band
Pair (1)	y=0.67x+58.60	y=0.66x+72.73	y=0.74x+40.40
Pair (2)	y=0.74x+50.13	y=0.69x+74.32	y=0.74x+59.31
Pair (3)	y=0.74x−1.20	y=0.93x+22.71	y=0.84x+94.08
Pair (4)	y=2.08x−335.61	y=1.92x−606.99	y=1.93x−680.19
Pair (5)	y=0.62x+189.02	y=0.81x+317.19	y=0.85x+332.35
Pair (6)	y=0.73x−50.21	y=0.73x−76.66	y=0.85x−133.18
Pair (7)	y=1.51x+21.56	y=1.46x+16.78	y=1.26x+65.70
Pair (8)	y=1.23x+9.97	y=1.51x−72.68	y=1.78x−171.29

**Figure 8:**
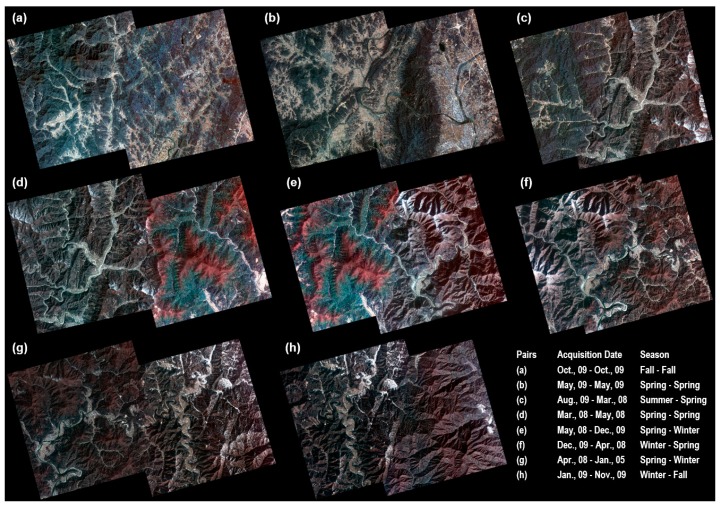
Final mosaic images for each pair of Kompsat-2 images. (**a**) Fall-Fall; (**b**) Spring-Spring; (**c**) Summer-Spring; (**d**) Spring-Spring; (**e**) Spring-Winter; (**f**) Winter-Spring; (**g**) Spring-Winter; (**h**) Winter-Fall.

**Figure 9:**
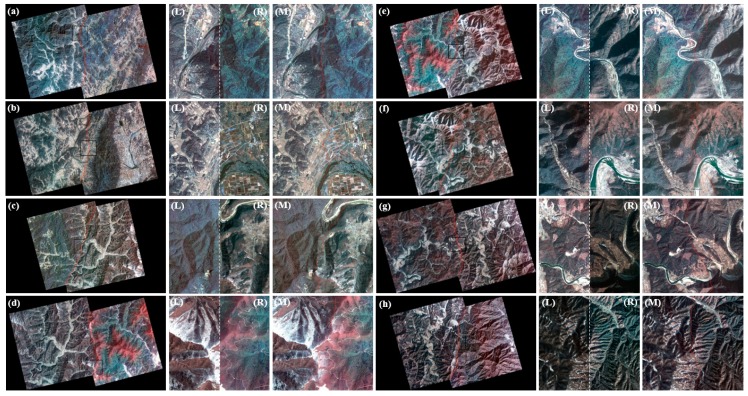
Mosaicing images before (*i.e.*, there are image color differences between the left and right images in the overlap area) and the after mosaicing images (*i.e.*, the color is adjusted over the entire area and the enlarged area). (**a**) Fall-Fall; (**b**) Spring-Spring; (**c**) Summer-Spring; (**d**) Spring-Spring; (**e**) Spring-Winter; (**f**) Winter-Spring; (**g**) Spring-Winter; (**h**) Winter-Fall; (**L**) Enlarged left area before mosaic; (**R**) Enlarged right area before mosaic; (**M**) Enlarged mosaic area.

Finally, eight mosaic images were created using the feathered images and the straight line equations (Figure 8). Figure 9 shows the resulting enlarged mosaics.

## 5. Performance Comparison

We have compared the mosaic image of the proposed algorithm with that of the previous algorithm, which has been widely used for an image mosaicing [15,16,17]. To evaluate a fair test, it is assumed that there are no additional works, such as the color balancing or enhancement. We have designed that the experimental conditions follow the procedure described in the Section 3. Table 5 summarizes the detailed experimental conditions used for this research.

**Table 5:** Experimental conditions used for performance evaluation.

Procedures	Algorithms
Input image	(see Figure 1g,h)
Masking image	(see Figure 5)
Extracting coordinates	(see Equation (1))
Extracting overlap area	$OA_{image}(x,y)=f1_{RGB}(x,y)\cap f2_{RGB}(x,y)$
Extracting seamline	Weighted seamline (geometry-based) [16,17]
Matching histogram	Band by band [15]
Feathering image	Distance: 200 pixels [17]
Output image	(see Figure 10)

**Figure 10:**
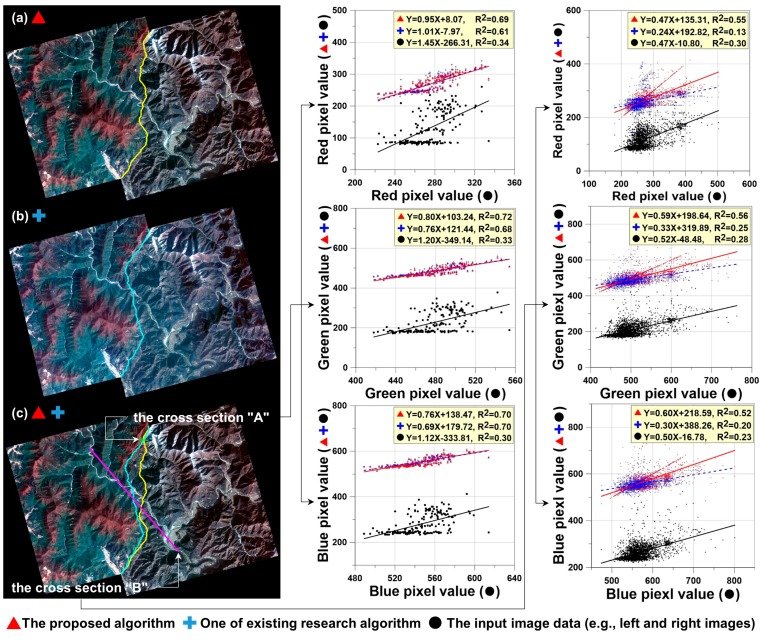
Comparisons between (**a**) the proposed and (**b**) previous algorithms. The red triangles denote the mosaic image by the proposed method, while the blue crosses indicate the mosaic image by the previous method. The black circles present the input image data. The yellow and blue lines in (**a**,**b**) show the seamlines from the proposed and previous methods, respectively. The graphs in the center and right lines are each generated from the cross-sections A and B of (**c**).

Our algorithm has been proposed to mosaic high-resolution images which show a distinct feature of extreme seasonality. Thus, to compare the proposed method with the previous one, the pair 5 of test pairs (see Figure 8e) was selected and used to demonstrate the comparative superiority of mosaic images. Figure 10 compares the mosaiced result between the proposed and previous methods. Figure 10a shows the mosaic image created by the proposed method while Figure 10b presents the mosaic image produced by the previous method. To compare the two mosaic images quantitatively, we have determined the cross-sections A and B, as shown in Figure 10c. Both the cross-sections A and B were extracted from the overlapped area. And the cross-section A was also extracted from the same feathering area in the proposed and previous methods. In the cross-sections, the brightness values of the original left image were considered as X variable’s values, while those of the original right, proposed mosaic and previous mosaic images were considered as Y variable’s values. Then we have calculated the linear fitting equations and *R^2^* values in the cross-sections A and B, respectively.

The cross-section A is used to evaluate the algorithm performance within the feathering area while the cross-section B is used for the performance evaluation over the whole area of the mosaic image. In the cross-section A, the *R^2^* for the proposed and previous mosaic images and the original images were (0.69, 0.72, 0.70), (0.61, 0.68, 0.70) and (0.34, 0.33, 0.30) in the red, green and blue bands, respectively. The proposed mosaic image is a little bit higher than the previous one (almost same), and both the mosaic images are almost two times higher than the original ones. It means that the performance of the proposed and previous methods are very similar and works well within the feathering area. In the cross-section B, the *R^2^* for the proposed and previous mosaic images and the original images were (0.55, 0.56, 0.52), (0.13, 0.25, 0.20) and (0.30, 0.28, 0.23) in the red, green and blue bands, respectively. The proposed mosaic image is two times higher than the original images while the previous mosaic image is a little bit lower than the original images. It indicates that our proposed method would be superior to the previous one over the whole area of the mosaic image. Moreover, the slopes of the linear fitting equations were (0.47, 0.59, 0.60), (0.24, 0.33, 0.30) and (0.47, 0.52, 0.50) in the red, green and blue bands, respectively. The proposed mosaic image is very close to the original images, but the previous mosaic image is apparently different from the original ones. It further validates that our proposed method would be better than the previous one.

## 6. Conclusions

In this study, a proposed mosaic procedure with limited assumptions was successfully tested using Kompsat-2 images. The conclusions from this study are summarized as follows: (1) based on this study, the proposed mosaic procedure can more efficiently produce high quality mosaic images using orthorectified and pan-sharpened Kompsat-2 images; (2) deciding the initial overlap area and extracting the final overlap area can affect the seamline decision, because the final overlap area reduces the whole overlap area; (3) the Sobel operator emphasizing edges can be used to produce an effective seam image and then a seamline can be extracted by iteratively choosing the minimum value from three adjacent pixels in the line direction without a threshold value; (4) color adjustment using a calculated straight line in the overlap area of two adjacent true color Kompsat-2 images is able to match the color of the mosaic image; and (5) although the Kompsat-2 images were obtained during different seasons, which can be observed in the differences in the colors of the overlap areas, the image feathering algorithm results in a natural mosaic image.

Based on the results of this research, we need more an in-depth study for an across-the-board mosaic in the foreseeable future, for instance, application of urban areas using various orthoimages (e.g., high-resolution optical satellite images, aerial photographs and orthoimages of two or more different types) and identical assumptions; influence on the horizontal and vertical seamline choices from seam image if the gradient magnitude image created using various kernel operators; the color adjustment using nonlinear equations calculated from overlap area of two or more adjacent images to match color of a mosaic image; determination of the efficient distance from center of the seamline when the feathering algorithm was used to create a mosaic image for more natural result; and finally, we need to establish a quantitative quality evaluation method for mosaic images.
